# Development of a Patient-Specific Multi-Scale Model to Understand Atherosclerosis and Calcification Locations: Comparison with *In vivo* Data in an Aortic Dissection

**DOI:** 10.3389/fphys.2016.00238

**Published:** 2016-06-21

**Authors:** Mona Alimohammadi, Cesar Pichardo-Almarza, Obiekezie Agu, Vanessa Díaz-Zuccarini

**Affiliations:** ^1^Mechanical Engineering, University College LondonLondon UK; ^2^Vascular Unit, University College HospitalLondon, UK

**Keywords:** mathematical modeling, multiscale, atherosclerosis, patient-specific, aortic dissection, *in vivo* data

## Abstract

Vascular calcification results in stiffening of the aorta and is associated with hypertension and atherosclerosis. Atherogenesis is a complex, multifactorial, and systemic process; the result of a number of factors, each operating simultaneously at several spatial and temporal scales. The ability to predict sites of atherogenesis would be of great use to clinicians in order to improve diagnostic and treatment planning. In this paper, we present a mathematical model as a tool to understand why atherosclerotic plaque and calcifications occur in specific locations. This model is then used to analyze vascular calcification and atherosclerotic areas in an aortic dissection patient using a mechanistic, multi-scale modeling approach, coupling patient-specific, fluid-structure interaction simulations with a model of endothelial mechanotransduction. A number of hemodynamic factors based on state-of-the-art literature are used as inputs to the endothelial permeability model, in order to investigate plaque and calcification distributions, which are compared with clinical imaging data. A significantly improved correlation between elevated hydraulic conductivity or volume flux and the presence of calcification and plaques was achieved by using a shear index comprising both mean and oscillatory shear components (HOLMES) and a non-Newtonian viscosity model as inputs, as compared to widely used hemodynamic indicators. The proposed approach shows promise as a predictive tool. The improvements obtained using the combined biomechanical/biochemical modeling approach highlight the benefits of mechanistic modeling as a powerful tool to understand complex phenomena and provides insight into the relative importance of key hemodynamic parameters.

## Introduction

Atherogenesis is a complex, multifactorial and systemic process; the result of a number of factors, each operating simultaneously at several spatial and temporal scales. The bewildering molecular and cellular complexity is well-described in Lusis' classical review more than a decade ago (Lusis, 2000), which highlights a plethora of biological mechanisms and gene associations, revealing an incredible etiological complexity. In addition to the biological components of the disease, atherosclerosis is also known to be related to mechanical stimuli on the vessel wall and hemodynamic parameters (Suo et al., 2006). Experimental evidence indicates that hemodynamic stimuli influence mechanotransduction and affect permeability (Davies, 1995). Increased permeability can lead to penetration and accumulation of lipoproteins (e.g., Low Density Lipoproteins–LDL) in the arterial wall and thus initiation of atherosclerosis. In recent years, much research has been conducted in order to draw correlations between hemodynamics and the atherogenic process (Peiffer et al., 2013a; Alimohammadi et al., 2015a). Certain hemodynamic parameters have been identified as key; these include flow distribution, pressure and wall shear stress (WSS) indices. Nevertheless, given the incredible complexity of the atherogenesis process, these hemodynamic analyses, on their own, have been inconclusive (Peiffer et al., 2013a) and a clear metric for plaque location remains elusive. Strong correlations between atherosclerotic disease and vascular calcification have been well-documented in the literature, including large cohort studies (Sangiorgi et al., 1998). Although the underlying molecular cause of calcification is unknown (Lanzer et al., 2014), the severity and extent of mineralization in calcification reflect atherosclerotic plaque burden (Demer and Tintut, 2008). Given the central role of inflammation in atherogenesis, an interesting possibility is that vascular mineral itself may initiate, promote, or perpetuate atherosclerosis by inducing inflammatory cytokines in monocytes that encounter and ingest hydroxyapatite crystals (Nadra, 2005).

Modeling and simulation have been used in a large number of studies in order to improve understanding of the role of hemodynamic variables in plaque formation. Although this work is mathematically elegant and can provide detailed insight into hemodynamics, it is disconnected from molecular research. Similarly, biomedical researchers often reduce the complexity of investigations of cardiovascular disease into manageable parts, for example, working on cell-lines or employing large-scale genome wide association studies (GWAS) to identify SNPs related to CVD (Tegner et al., 2006). However, such statistical genetic models have no mechanistic basis, and it is significant that Lusis highlighted the revival of functional studies in a relatively recent review (Lusis, 2012).

In this paper, a clear application of mathematics for healthcare will be made by unifying multi-mechanistic factors in the prediction of atherosclerosis location. The location of atherosclerotic plaque and vascular calcification will be investigated using a patient-specific biomechanical model of an aortic dissection (AD). From a physiological point of view, AD is a life threatening condition in which a tear forms in the wall of the aortic wall and blood splits the media layer, forming two lumina: the true lumen (TL) and false lumen (FL; Braverman, 2010). Blood flows from the TL into the FL via a primary tear and, in communicating dissections, returns to the TL via one or multiple tears downstream. The section of intima and media that separates the two lumina, called the intimal flap (IF), often stiffens over time due to fibrosis (Criado, 2011). Common comorbidities in patients with AD are atherosclerosis (Coady et al., 1999; Tsai et al., 2006) and inner wall calcification (de Jong et al., 2014). Additionally, patients suffering from AD usually have an elevated pulse pressure, which would likely be further increased in the presence of calcification, due to the reduction of vessel elastance (Demer and Tintut, 2008). The patient data used for this research showed an AD with significant atherosclerosis and calcification regions, consistent with the condition. Detailed hemodynamic characterization was achieved by using patient-specific dynamic boundary conditions representing the downstream vasculature, based on *in vivo* measurements collected for the same patient, treated in University College Hospital (UCH; Alimohammadi et al., 2014). Although complex flow simulations for this patient have been published previously, the work presented here, including the quantification and analysis of plaque and calcification areas as well as the multi-scale framework used in this context, is completely new.

The postulate of this research is that multiscale modeling and simulation can pave the way to study multi-mechanistic factors to explain disease in a cohesive modeling framework, which can integrate key markers at different biological scales and can provide insight into endothelial mechanotransduction, as well as potential predictive power in patient-specific analyses, compared to purely hemodynamic, biomechanical or biochemical approaches. We will use a virtual “follow-up” approach, combining a fluid-structure interaction (FSI) simulation model of a dissected aorta with a model of plaque formation, using a number of somewhat disparate indicators available in the literature, which will be described below. The patient-specific simulation results are then coupled to an endothelial permeability model following the three-pore approach (Olgac et al., 2008a), also recently used by Kim and Giddens (2015).

This paper is organized as follows: Section Methods presents the methods and simulation details. Results are presented in Section Results, including a clear, interpretable metric for atherogenic potential, which shows a better performance when compared to others based on “established” descriptors in the current literature. Simulation results of the model will be presented in and compared to atherosclerotic plaques and calcifications indicated in the original CT scans. The discussion, limitations and conclusions of this work will be presented in Section Discussion.

## Methods

### Details of the simulation

The specifics of the FSI simulation used for the present study are presented briefly in this section and are described in detail in Alimohammadi et al. (2015b). The patient gave informed oral consent and ethical approval was given by the National Research Ethics Service, UK, REC reference: 13/EM/0143. The fluid domain was reconstructed from CT scans of a female patient, starting at the ascending arch and extending down to the thoracic aorta, upstream of the iliac bifurcation. The supra-aortic branches were included in the model, but the visceral branches were not clearly resolvable from the CT data and so were omitted. The vessel wall was modeled by extruding the outer wall of the fluid geometry uniformly by 2.5 mm, based on reports of thickened aortic walls in hypertension (Malayeri et al., 2008), which occurs in ~70% of AD patients (Hagan et al., 2000; Khan and Nair, 2002). The intimal flap (IF), separating the TL and FL, was created by filling the gap between the two lumina and was 2.45 ± 0.34 mm (median ± median absolute deviation). Fluid and solid meshes were generated in ANSYS ICEM-CFD, and contained ~230,000 (with 7 prismatic layers at the wall) and 50,000 elements respectively.

Simulations were carried out using ANSYS mechanical and CFX. The vessel wall was modeled using the isotropic hyperelastic model of Raghavan and Vorp (2000). This model is comparable to a linear elastic model with a Young's modulus of 1 MPa, but displays a small amount of strain-stiffening. An external pressure of 52.5 mmHg (diastolic pressure in the descending aorta) was applied and at each of the outlets of the 3D domain, the solid geometry was restricted to planar motion about the center-point of the lumen, enabling expansion of the vessels.

At the inlet, a flow wave from another study of a patient with type-B aortic dissection was used (Karmonik et al., 2008), as such data was not available for the present patient. At the fluid outlets, three element Windkessel models were used, with parameters tuned to invasive pressure measurements on the patient, using an iterative technique described in a previous study (Alimohammadi et al., 2014). Blood was modeled as an incompressible fluid of density 1056 kg/m^3^. Turbulence was modeled using the hybrid k-ε, k-ω shear stress transport (SST) turbulence model, with a 1% turbulence level at the inlet. In order to account for the shear-thinning properties of blood, the Carreau-Yasuda (CY) viscosity model was utilized with the parameters reported by Gijsen et al. (1999). The CY viscosity model includes infinite and zero shear viscosities, a characteristic time constant and two exponents without direct physical meaning to describe the viscosity-shear response of blood. The parameters of Gijsen et al. (1999) were fitted to a blood analog, which showed similar characteristics to blood samples. The viscosity predicted by this model is ~3.5 mPa s (a commonly used Newtonian viscosity) at 100/s, and decreases for higher shear rates to around 2.5 mPa s at 1000/s. At shear rates below 100/s, the viscosity increases, exceeding 10 mPa s for shear rates below 2/s.

Simulations were run with a time step of 5 ms, a periodic solution was achieved after two cycles and the third cycle was extracted for further analysis. In a previous study (Alimohammadi et al., 2015b), it was shown that the low shear rates in the slow flow regions of the distal and proximal FL led to significantly increased viscosity.

### Shear stress indices (SSI)

In the permeability calculations described in Section Endothelial Permeability Model, one of the inputs is an index indicative of the shear stress condition that leads to increased permeability. The time-average wall shear stress (TAWSS) and oscillatory shear index (OSI; Ku et al., 1985) are two commonly used indices that are considered important for plaque formation. TAWSS describes the average magnitude of the shear stress and the OSI gives an indication of the directionality of the shear stress, yielding 0 for uniaxial flows and 0.5 when there is no preferential direction. Typically, in permeability models for atherogenesis, TAWSS is used as the shear index related to permeability.

A number of studies (Malek et al., 1999; Xiang et al., 2010; Chiu and Chien, 2011; Meng et al., 2014) have shown that regions with low average shear stress *combined* with highly oscillatory shear stress have increased endothelial permeability along with other pathological responses. More recently, Sáez et al. (2015) presented the 3D remodeling of endothelial cells as the combined effect of OSI and TAWSS in a computational framework, which fitted experimental works presented before in *in vitro* studies.

In light of the indications for increased permeability in low, oscillatory regions, we propose an index, HOLMES (Highly Oscillatory, Low MagnitudE Shear), given by:

(1) $$\begin{array}{r} HOLMES\;=\;TAWSS\left(0.5-OSI\right) \end{array}$$

This parameter is equivalent to half the reciprocal of relative residence time (RRT) which was previously identified as a potential index for combining these two characteristics (Himburg, 2004). The *HOLMES* indicator can be understood as a modified *TAWSS*, with the (0.5 − *OSI*) term further reducing the index in regions where the wall shear stress is both low in magnitude and oscillatory in nature. Additionally, HOLMES provides a conceptually alternative explanation, offering a linear (rather than reciprocal) index, proportional to WSS that intuitively corresponds to the observed effects of shear characteristics on endothelial permeability.

In the present study, we compare the efficacy of using the most-widely used hemodynamic/shear stress index (SSI) for atheroprone regions, i.e., TAWSS, with HOLMES, separate and in a mechanistic model, as shown in the sections below.

### Endothelial permeability model

The endothelial permeability model proposed here is based on previous work describing the early stages of atherosclerosis, using a transport model of low density lipoprotein (LDL) from the artery lumen into the arterial wall, taking into account the effects of mechanical stimuli exerted by the blood flow on the endothelial cell layer and its pathways of volume and solute flux; see Díaz-Zuccarini et al. (2014) for more details. An excellent and recent analysis and use of this model along the same lines using time-average wall shear stress (TAWSS) has been recently published by Kim and Giddens (2015).

The endothelial layer is described with a three-pore modeling approach considering the contributions of the vesicular pathway, normal junctions, and leaky junctions. The fraction of leaky junctions is calculated as a function of the mechanical stimuli and is used in conjunction with the pore theory to determine the transport properties of this pathway.

The LDL transport equations are decomposed using three main penetration pathways: leaky junctions, normal junctions and vesicular pathways; so the bulk of volume flux (*J*_*v*_) through the endothelial membrane is given by:

(2) $$\begin{array}{r} J_{v}\;={\;J}_{v,lj}\;+{\;J}_{v,nj} \end{array}$$

where *J*_*v, lj*_ is the flux through leaky junctions and *J*_*v, nj*_ is the flux through normal junctions.

The volumetric flux through leaky junctions (*J*_*v, lj*_) is calculated using a modified version of the Kedem-Ketchalsky equations for membrane transport:

(3) $$\begin{array}{r} {\;J}_{v,lj}\;={\;L}_{p,lj}\;\left(\Delta p_{end}-\sigma_{d}\Delta \Pi \right) \end{array}$$

where *L*_*p, lj*_ is the hydraulic conductivity, Δ*p*_*end*_ is the pressure difference through the endothelium, σ_*d*_ is the osmotic reflection coefficient and ΔΠ is the osmotic pressure. The value of Δ*p*_*end*_ is estimated by subtracting the externally applied pressure from either the average pressure throughout the domain (uniform pressure gradient) or the spatially varying time average pressure at each wall location (time-averaged pressure gradient).

According to the three *pores* theory, solute flux (LDL flux in this case) only occurs through endothelial leaky cell junctions and vesicles:

(4) $$\begin{array}{r} J_{s}\;={\;J}_{s,lj}\;+{\;J}_{s,v} \end{array}$$

Assuming that the solute flux through the vesicular pathway (*J*_*s, v*_) is 10% of the solute flux through the leaky junction pathway (*J*_*s, lj*_) (Olgac et al., 2008b).

Finally, in the interest of brevity, Equation (5) shows a general form of the function used to calculate *J*_*s, lj*_ which is proportional to the magnitude of *J*_*v, lj*_ (more details about the calculation of *J*_*s, lj*_ are shown elsewhere (Díaz-Zuccarini et al., 2014; Alimohammadi et al., 2015a):

(5) $$\begin{array}{r} {\;J}_{s,lj}\;=\;\phi \left({\;J}_{v,lj},Pi,Pe,\sigma ,{\;c}_{lum},\;{\;c}_{w,end},{\;c}_{ave}\;\right) \end{array}$$

where *Pi* is the diffusive permeability, *Pe* the modified Peclet number, *c*_*lum*_ and *c*_*w, end*_ the LDL concentration in the lumen and the sub-endothelial layer respectively, *c*_*ave*_ is the mean endothelial concentration and σ the solvent drag coefficient.

Endothelial cell shape will affect the amount of leaky junctions. Experimental findings have shown that in areas of altered hemodynamics, endothelial cells do not have a typical cobblestone shape, but rather exhibit a more circular shape as well as increased permeability. Previous models used a relationship between endothelial permeability and local WSS based on the Endothelial Cell Shape Index (ECSI; Levesque et al., 1986). ECSI is related to the cellular shape and takes values from zero to one, i.e., a circle has an ECSI of one whilst a straight line has an ECSI of zero.

Some approaches based on ECSI calculated this variable as a function of WSS (for instance Olgac et al., 2008b used WSS values in steady state simulations). However, as previously mentioned, recent work shows that ECSI can be affected by other indices *as well*, such as OSI. Based on the seminal paper by Levesque et al. (1986), excellent work from Sáez et al. (2015), presents how different OSI and TAWSS modify the endothelial cell shape. This combined relationship will be key for the modeling work presented here.

In the present study, we consider the role of the two different shear stress indices (SSI), i.e., TAWSS and HOLMES, described in Section Shear Stress Indices (SSI).

For each SSI, *ECSI* is defined according to

(6) $$\begin{array}{r} ECSI\;=\;0.380e^{-0.79SSI}\;+\;0.225e^{-0.043SSI} \end{array}$$

**Figure 1:**
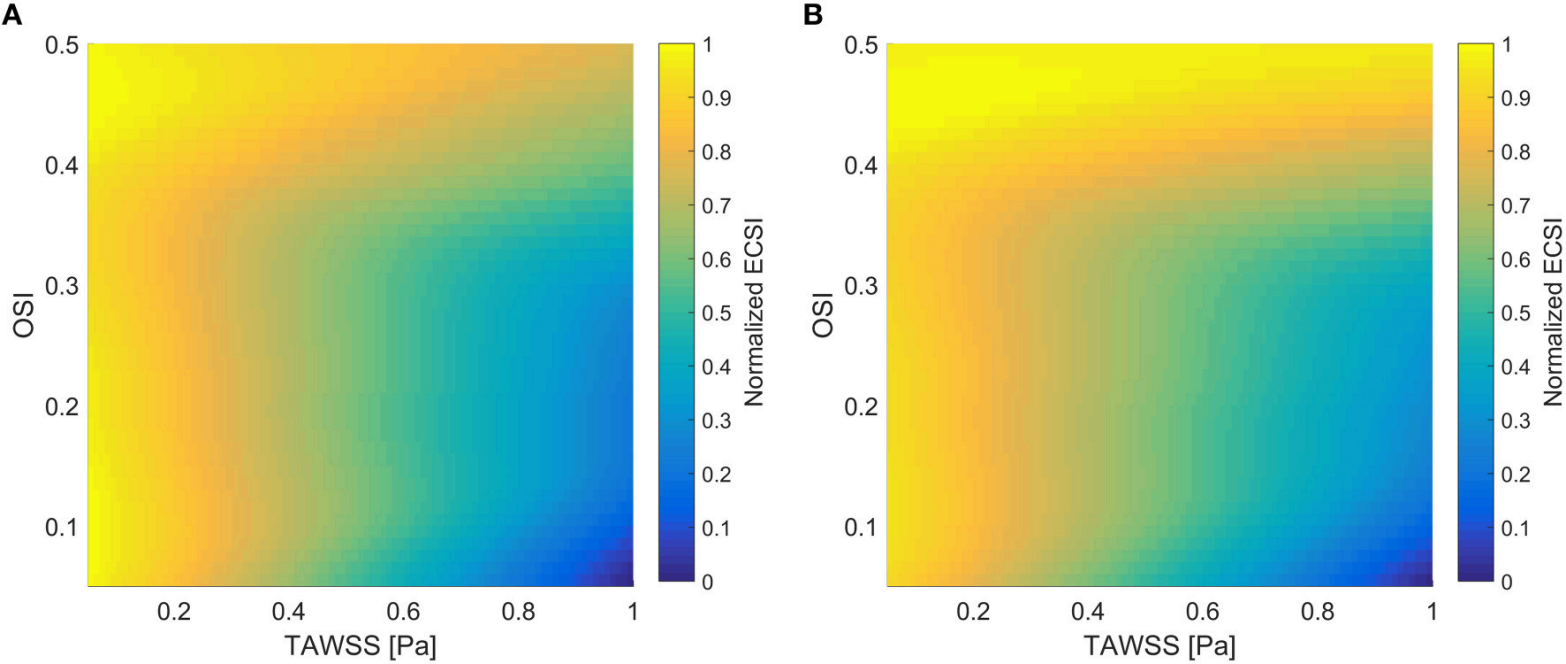
**Normalized ECSI as a function of TAWSS and OSI**. **(A)** Using Saez et al. results (Sáez et al., 2015); **(B)** Using Equation (6) and HOLMES.

Figure 1 shows a comparison of normalized ECSI using Saez et al. results (Sáez et al., 2015), which are dependent on TAWSS and OSI, with the normalized ECSI using Equation (6) and HOLMES. Very similar behavior can be seen in both surfaces, showing how areas of low TAWSS and high OSI will increase the values of ECSI. Following this, we will use HOLMES as a combined index able to capture key haemodynamic features in the regions of interest for atherosclerotic plaque/calcification locations.

Leaky cells have high permeability to LDL, which can be linked to the magnitude of the SSI. Areas with high ECSI will be related to a higher number of mitotic cells (*MC*) which are calculated as follows:

(7) $$\begin{array}{r} MC\;=\;0.003739e^{14.75ECSI} \end{array}$$

Assuming that within the endothelium the quantity of leaky mitotic cells is ~80.5%, which represents ~45.3% of the total number of leaky cells (*LC*) in that area (Tedgui and Lever, 1984a), the number of *LC* is calculated as:

(8) $$\begin{array}{r} LC\;=\;0.307\;+\;0.805MC \end{array}$$

The ratio of endothelium (ϕ) covered by LCs is calculated using

(9) $$\begin{array}{r} \phi =\frac{LC\;\pi R_{cell}^{2}}{unit\;area} \end{array}$$

where *R*_*cell*_ is the radius of a single cell. Finally, the total hydraulic conductivity of the endothelial leaky junctions (*L*_*p, lj*_) is defined as:

(10) $$\begin{array}{r} L_{p,lj}=\phi \cdot L_{p,slj} \end{array}$$

where *L*_*p, slj*_ is the hydraulic conductivity of a single leaky junction calculated as follows:

(11) $$\begin{array}{r} L_{p,slj}=\frac{w^{2}}{3\mu l_{lj}} \end{array}$$

with *w* and *l*_*lj*_ being the half-width (20 nm) and the length (2 μm) of the leaky junctions and μ the viscosity term used for the estimation of the LDL penetration.

### Estimating plaque location and metrics evaluation

**Figure 2:**
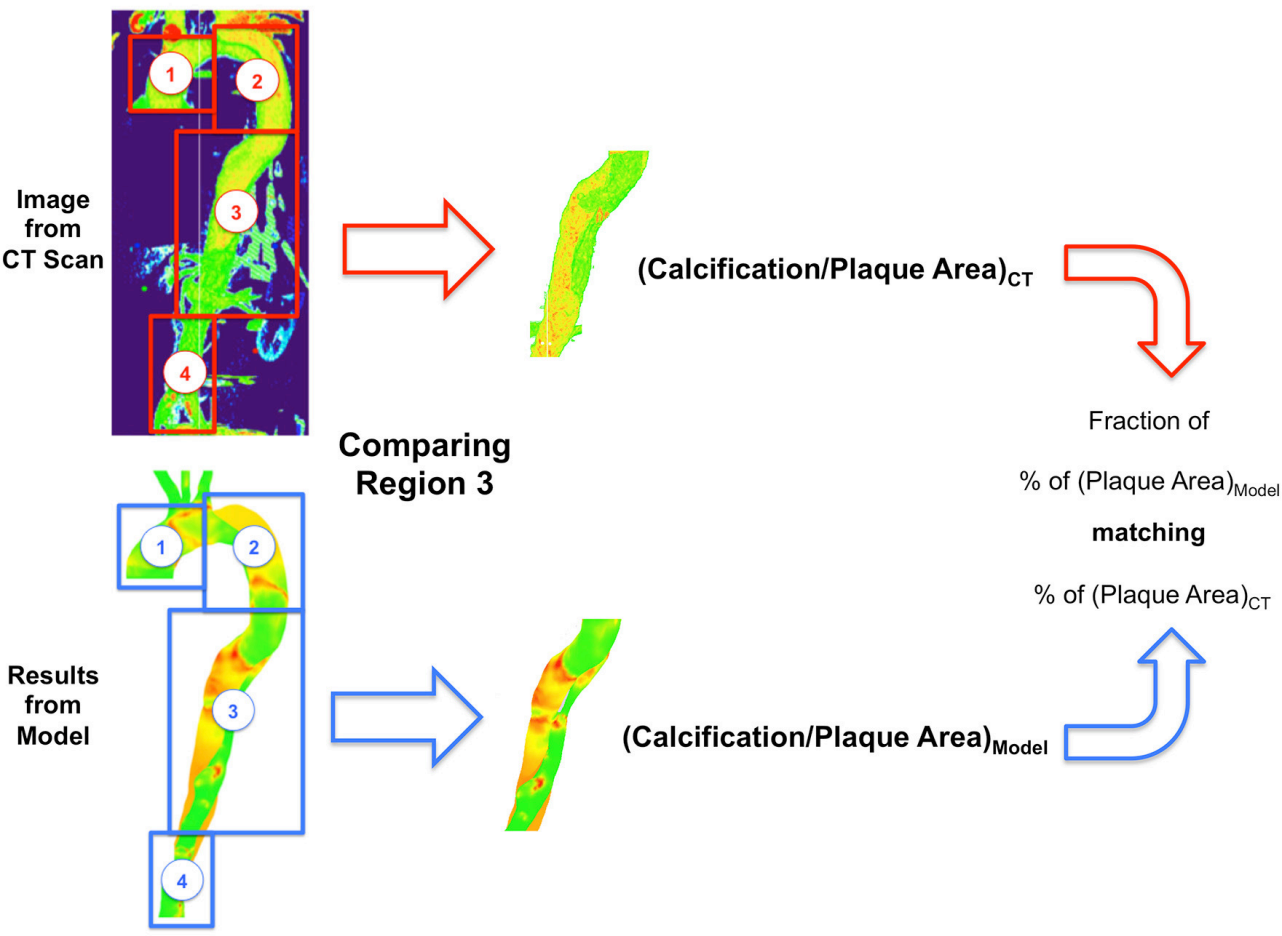
**Definition of the different regions in the aorta for comparison and analysis**.

Previous work (Alimohammadi et al., 2015a) has shown that calculation of the hydraulic conductivity in leaky junctions (*L*_*p, lj*_) can be used to estimate the magnitude of LDL fluxes across the wall along the artery and identify atheroprone regions. However, as mentioned previously, other variables such as the volume flux *J*_*v, lj*_ [which is closely related to the solute (LDL) flux, *J*_*s, lj*_], may be a better metric to identify atheroprone areas. As shown in Equation (3), *J*_*v, lj*_ is proportional to both *L*_*p, lj*_ and the pressure drop across the endothelium Δ*p*_*end*_ and thus, *J*_*v, lj*_ may be more sensitive in the estimation of plaque locations. For a given metric (*J*_*v, lj*_ or *L*_*p, lj*_), the aorta was divided into subsections and the mapping of this variable was compared with the plaque/calcification observed in the same subsections from CT scans, as shown in Figure 2. Plaque areas are calculated as a percentage (%) of the total area covered for the selected section of the artery, with plaque defined as HU>220 in the clinical image (Isgum et al., 2004) and *L*_*p, lj*_ >1.2 × 10^−11^ m^2^s/kg (Tedgui and Lever, 1984b). The ratio of the area coved in the model compared to the clinical image is reported as a percentage match.

Comparison of the calcification/plaque area observed in the CT scans and those calculated by the chosen metric following the methodology shown here, was used to evaluate the efficacy of the different metrics and the role of some of the selected hemodynamic (viscosity and SSI) variables.

## Results

**Figure 3:**
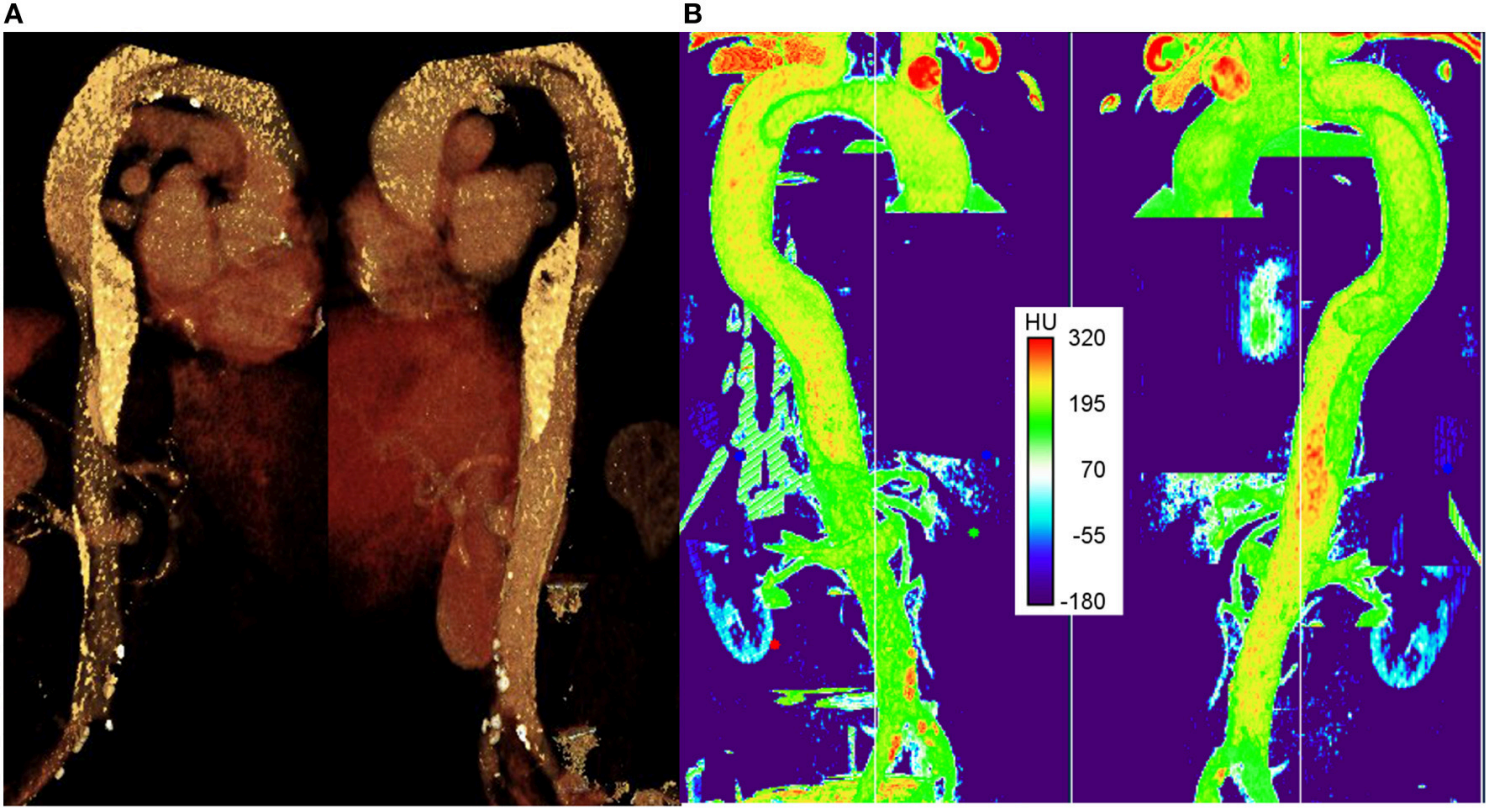
**The right anterior and left posterior views of the aorta**. **(A)** 3D CT data visualized in Aquarius and **(B)** 3D LUT volume viewer in FIJI (Image J).

Figure 3A shows an image of the 3D CT image reconstruction in Aquarius (TeraRecon, USA), in which the highest intensity regions indicate plaques (white) or vessel wall calcification. Figure 3B shows an image of the CT scans reconstructed in the LUT volume viewer in Fiji (Schindelin et al., 2012), with intensities in the range 0–255. The colorbar shows Hounslow units (HU) from the CT scan. Although there is not a single threshold that can be considered to be a calcification, a value of 220 HU has been used previously as an indicator of calcified regions (Isgum et al., 2004). The plaques appear red and the calcifications yellow (it should be noted that the exact rendering of both panels in Figure 2 is dependent on the distance from the first slide as there is no easy way to ensure the same depth in both pieces of software, and so the views may differ slightly).

As seen in both panels, a number of completely formed plaques can be observed in the lower edge of the aortic arch, between the two tears in FL and around the iliac bifurcation. Calcifications can be seen along the aortic arch proximal to the supraaortic branches, and in the proximal and distal FL. The existence and locations of the plaques and calcifications were confirmed and approved by the vascular surgeon who managed this patient.

### Wall shear stress indices

**Figure 4:**
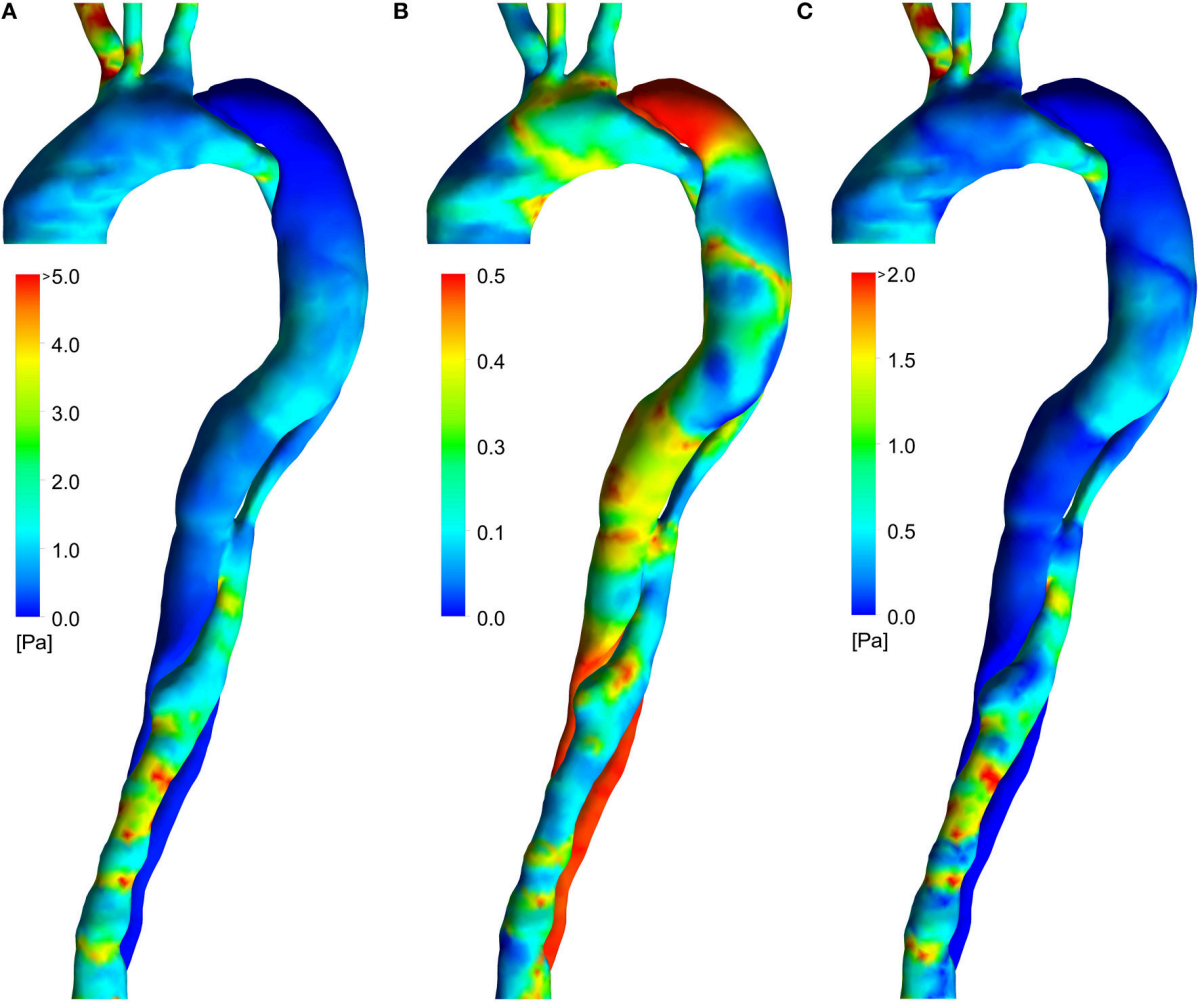
**Wall shear indices from the FSI simulations**. **(A)** TAWSS, **(B)** OSI, **(C)** HOLMES.

Figure 4 shows the distributions of the three SSI used in the present study. The TAWSS (Figure 4A) is moderate in the ascending aorta and in the FL between the two tears. Elevated regions of TAWSS can be observed in the visceral branches, at the coarctation and in the distal TL. The OSI (Figure 4B) shows scattered elevated regions throughout the domain, with particularly high values in the distal and proximal FL (which would not be captured using a rigid wall model; Alimohammadi et al., 2015b). The HOLMES index (Figure 4C) is similar to TAWSS, but lower in magnitude overall, and particularly in regions where the OSI is high, such as between the two tears and in the aortic arch.

In the following sub-sections, we systematically compare the indices and parameters used in the literature to indicate or predict locations of calcification and/or atherogenesis with our integrated model, the use of HOLMES and spatially varying viscosity.

### Analysis of the total hydraulic conductivity

As the volume flux is dependent on both hydraulic conductivity and pressure gradient across the endothelium, we first consider *L*_*p*_ alone.

**Figure 5:**
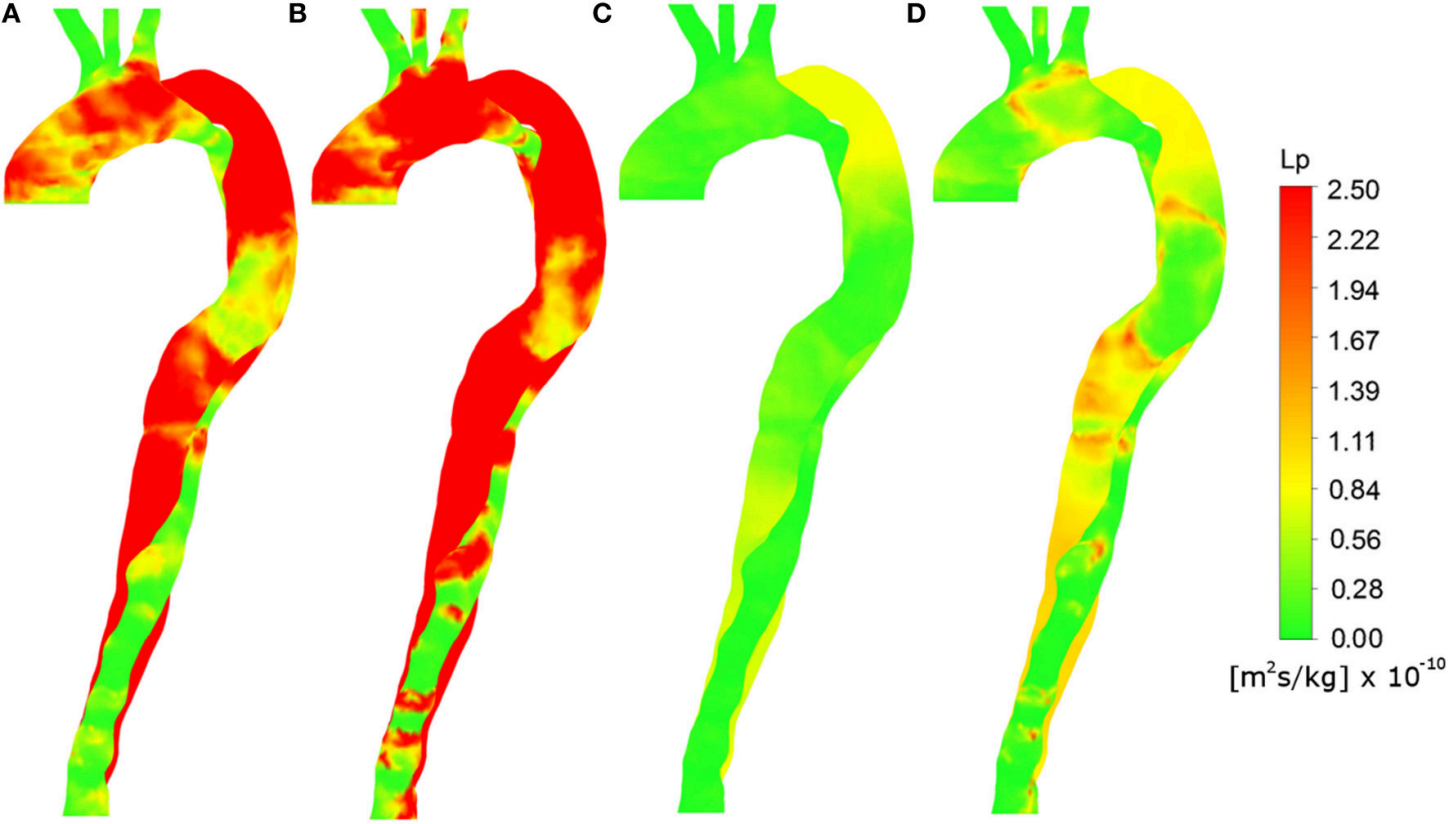
**Total hydraulic conductivity of leaky junctions (***L***_***p, lj***_) values calculated using various parameter combinations from the FSI results shown in the right anterior view**. **(A)** Constant plasma viscosity and TAWSS, **(B)** Constant plasma viscosity and HOLMES, **(C)** Non-Newtonian blood viscosity and TAWSS, **(D)** Non-Newtonian blood viscosity and HOLMES.

Figures 4A,B show the total hydraulic conductivity of leaky junctions (*L*_*p, lj*_) values calculated assuming a constant viscosity at the wall, equal to the plasma viscosity and TAWSS and HOLMES, respectively, as wall shear stress indices. Figures 4C,5D show the same wall shear stress indices, but with the viscosity at the wall predicted based on the continuum assumption using the Carreau-Yasuda viscosity model. Note that all panels use the same color scale. Tedgui and Lever (1984b) reported *L*_*p, lj*_ values of 1.2 × 10^−11^ m^2^s/kg, corresponding to green values in the figure. Higher values indicate pathologically elevated *L*_*p, lj*_. For both of the plasma viscosity models (Figures 4A,B), the estimated *L*_*p, lj*_ values are extremely high, and no qualitative correlation can be observed when comparing these images to Figure 3B. Using the non-Newtonian viscosity model at the vessel wall and TAWSS to calculate *L*_*p, lj*_ (Figure 5C), reduced the magnitude of the calculated *L*_*p, lj*_ and resulted in a reasonable indication of the calcified/atheroprone locations in both the distal and proximal FL, although the extent of the calcified regions is shorter than those observed in Figure 2 and the calcified region between the two tears is not identified. Additionally, this model did not identify any of the plaque locations. Comparing the *L*_*p, lj*_ distribution predicted using HOLMES and non-Newtonian viscosity (Figure 5D) to the clinical images (Figure 3B), it can be seen that there is a good qualitative correlation between the two figures. Figure 5 shows this *L*_*p, lj*_ distribution in greater detail in both right anterior and left posterior views.

**Figure 6:**
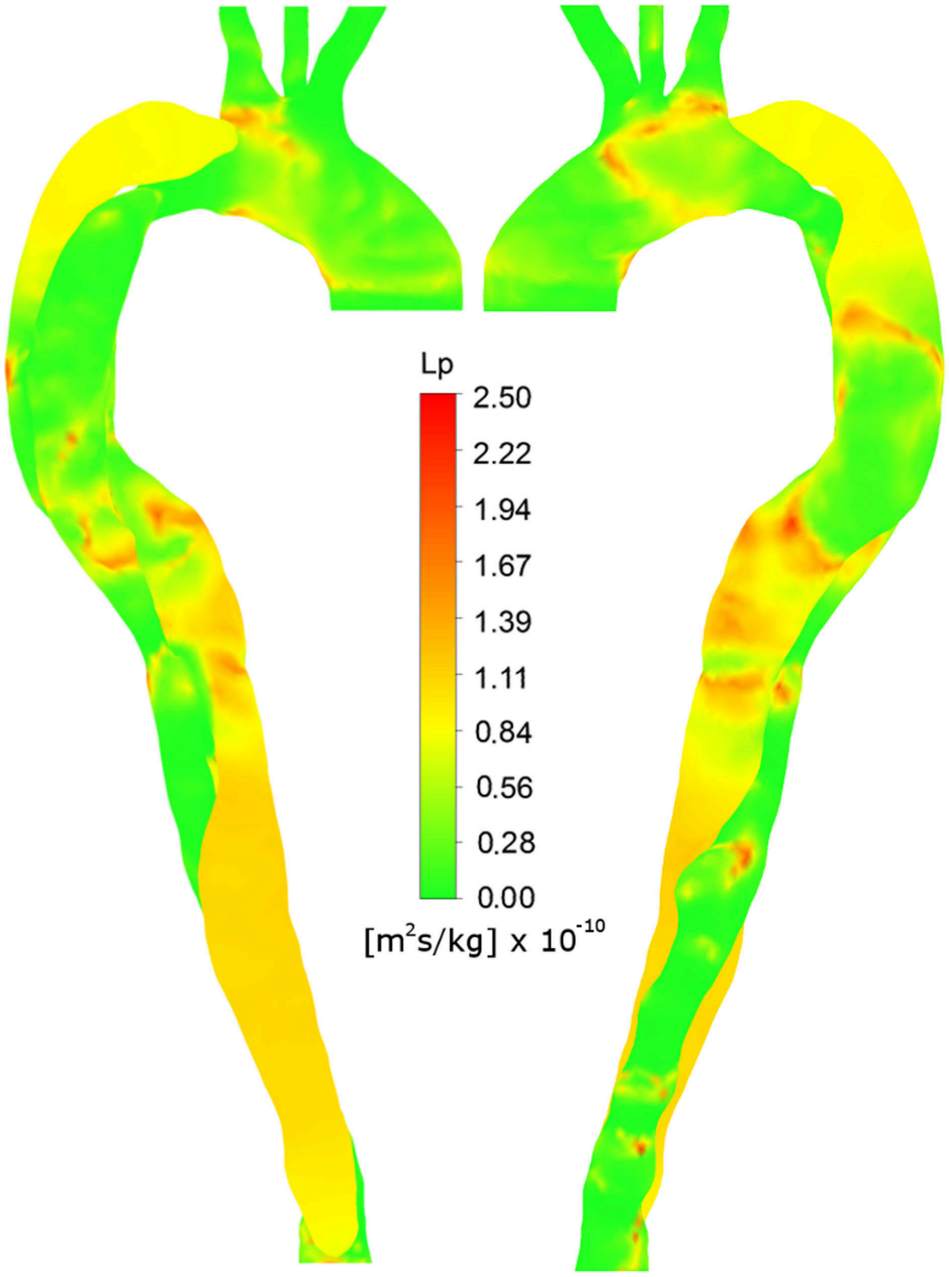
**Right anterior and left posterior views of ***L***_***p, lj***_, calculated using non-Newtonian viscosity and HOLMES**.

Under the assumption that plaque formation and calcification occur as a result of elevated *L*_*p, lj*_, the correlation between Figures 6, 3B implies that this combination of variables is able to identify regions of calcification and plaque formation, and thus could be potentially used to predict further development of such pathologies.

The region of calcification with scattered small plaques observed in the FL between the two tears is captured in the *L*_*p, lj*_ prediction in Figure 6. Similarly, the calcification of the proximal FL (both views) and the distal FL (left posterior view), are predicted by elevated *L*_*p, lj*_ in these regions. The low *L*_*p, lj*_ in the distal TL and ascending arch in the left posterior view also correlate with minimal calcification in Figure 3B. The plaques at the lower edge of the aortic arch, best observed in Figure 3A in the left posterior view, correspond to regions of elevated *L*_*p, lj*_ in Figure 6.

Some regions of the image do not correlate, such as the low *L*_*p, lj*_ in the right anterior view of the ascending arch, where there is calcification in the CT image.

### Analysis of the volume flux

Figure 6 provides images of the predicted volume flux across the vessel wall, *J*_*v, lj*_, using various combinations of wall shear indices and viscosity assumptions, along with either a uniform pressure gradient or a time-averaged pressure gradient. These figures can be compared with Figure 3B to analyze the hypothesis that calcification and plaque formation are better correlated with volume flux, *J*_*v, lj*_, rather than *L*_*p, lj*_ alone. Meyer et al. (1996) reported *J*_*v*_ values in the range ~2–3 × 10^−8^ m/s, corresponding to green regions in the figure. Higher values indicate elevated solute flux.

**Figure 7:**
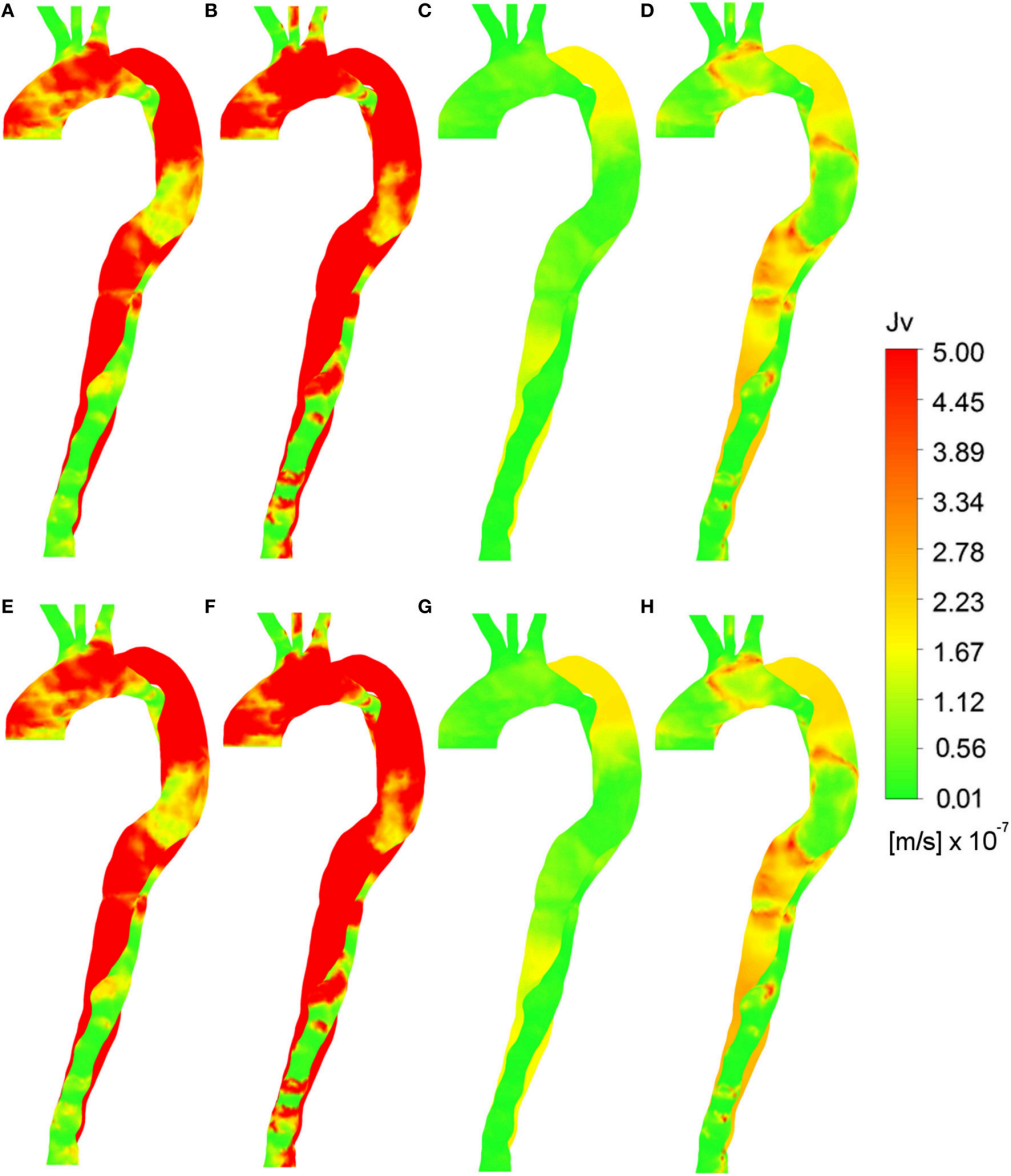
**Volume flux (***J***_***v, lj***_) calculated using various parameters**. Wall shear indices **(A)** TAWSS, **(B)** HOLMES with constant plasma viscosity and uniform pressure gradient. Wall shear indices **(C)** TAWSS, **(D)** HOLMES with non-Newtonian blood viscosity and uniform pressure gradient. **(E)** TAWSS, **(F)** HOLMES with constant plasma viscosity and time averaged pressure gradient. **(G)** TAWSS, **(H)** HOLMES with non-Newtonian blood viscosity, and time-averaged pressure gradient.

**Figure 8:**
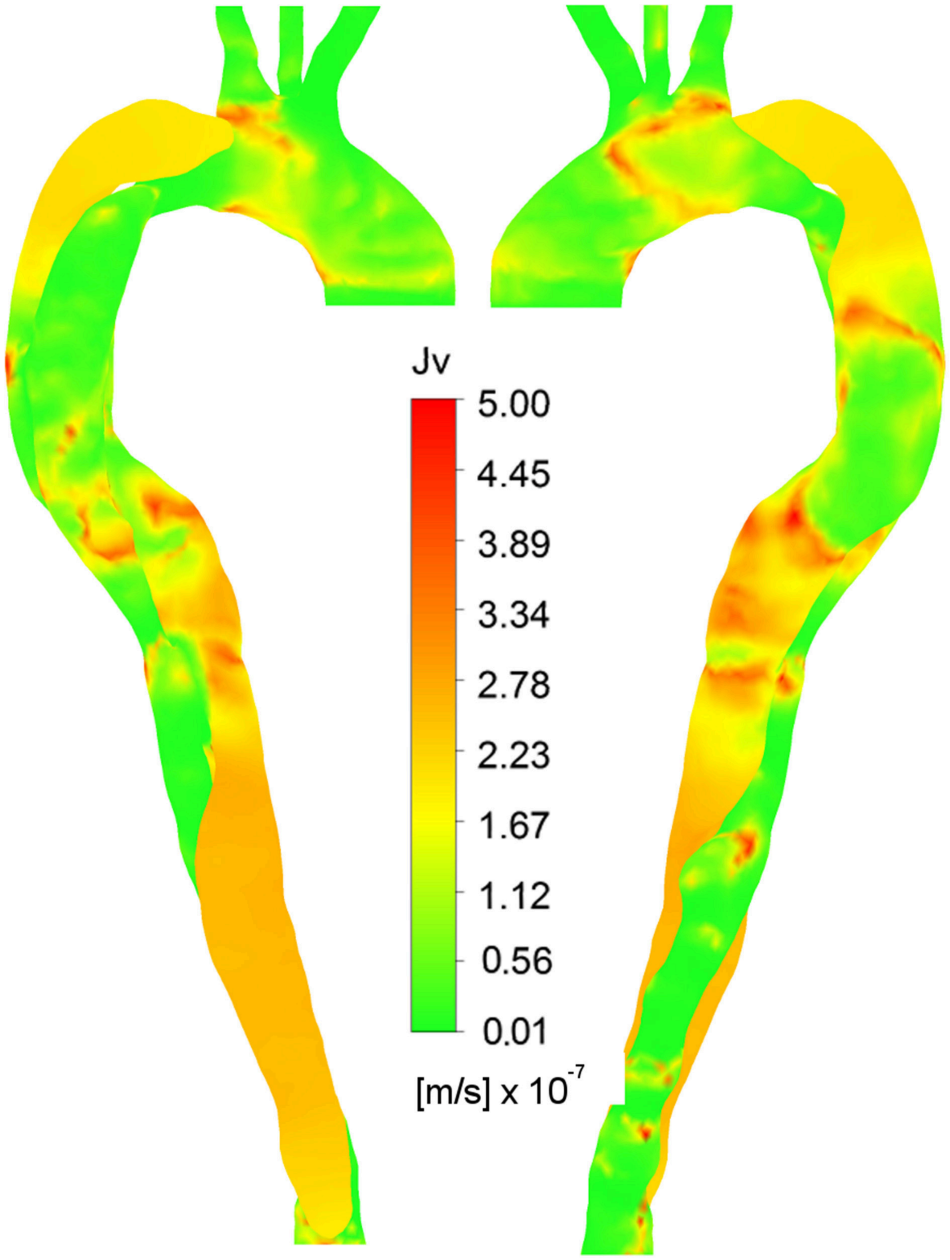
**Right anterior and left posterior views of ***J***_***v, lj***_, calculated using non-Newtonian viscosity, HOLMES and time-averaged pressure gradient**.

None of the constant plasma models (Figures 7A,B,E,F produced *J*_*v, lj*_ distributions that compared well with Figure 3B. Figures 7C,D using the uniform pressure gradient are qualitatively identical to Figures 4C,D as the uniform pressure distribution does not affect the relative values of *J*_*v, lj*_ as compared to *L*_*p, lj*_. When using the time averaged pressure gradient (Figures 7G,H), the distributions do not significantly change in qualitative terms, indicating that the prediction is more sensitive to *L*_*p, lj*_, and thus appropriate models for wall shear stress and blood viscosity at the wall, than to the pressure gradient. Figure 8, showing the distribution of *J*_*v, lj*_ using HOLMES, the non-Newtonian model and time averaged pressure distribution, is therefore qualitatively similar to Figure 6, although there are some differences in the magnitudes.

## Discussion

The results shown in Section Results are promising and thus warrant further investigation. They also open up a number of questions. Is the volume flux (which determines the magnitude of the solute flux) a metric for plaque location? If so, then what is required in order to estimate it correctly? As mentioned in the introduction, the question of plaque location remains elusive. There are presently no available tools to predict where this might occur, or indeed to sufficiently explain why. This may be because, if we take volume flux (*J*_*v, lj*_) (or even hydraulic conductivity) as the metric for plaque location, its generation is multifactorial and complex and thus simplified models lack some of the key features that seem to impact plaque location (see Section Results). In the present study, strong correlations were only produced through the use of a multiscale model combining multiple hypotheses at the cellular and physiological level, as evidenced by the comparison of Figures 5, 7 with Figure 3. This complex approach involves the detailed formulation, extraction and combination of more than 11 different variables including the calculation of variable pressure differences along the arterial wall across the endothelium, the use of a non-linear model for blood viscosity and use of the HOLMES index to isolate regions of low, oscillatory shear. This formulation, making use of mechanistic models, also provides these metrics (*J*_*v, lj*_ and *L*_*p, lj*_) with strong interpretability and physical meaning. As previously explained, the origin and interpretation of HOLMES as a combined index is strongly rooted in the most recent, relevant literature (Sáez et al., 2015). The work presented in this paper exemplifies the power of mechanistic models as “hypothesis tester” and a key example of the use of mathematics and mechanistic models and formulations *to understand* biological processes. In fact, it can be can argued that *a posteriori hypotheses*, as a result of abandoning part of a priori thinking in the light of new observations, can pave the way for future studies (Erren, 2007). Here, an unambiguous formulation could enable others to follow up on the findings and modified conjectures to advance knowledge in this field. Despite some limitations (please see below), it remains a fact that the work presented here is strongly anchored in the state of the art and current knowledge in this area.

**Figure 9:**
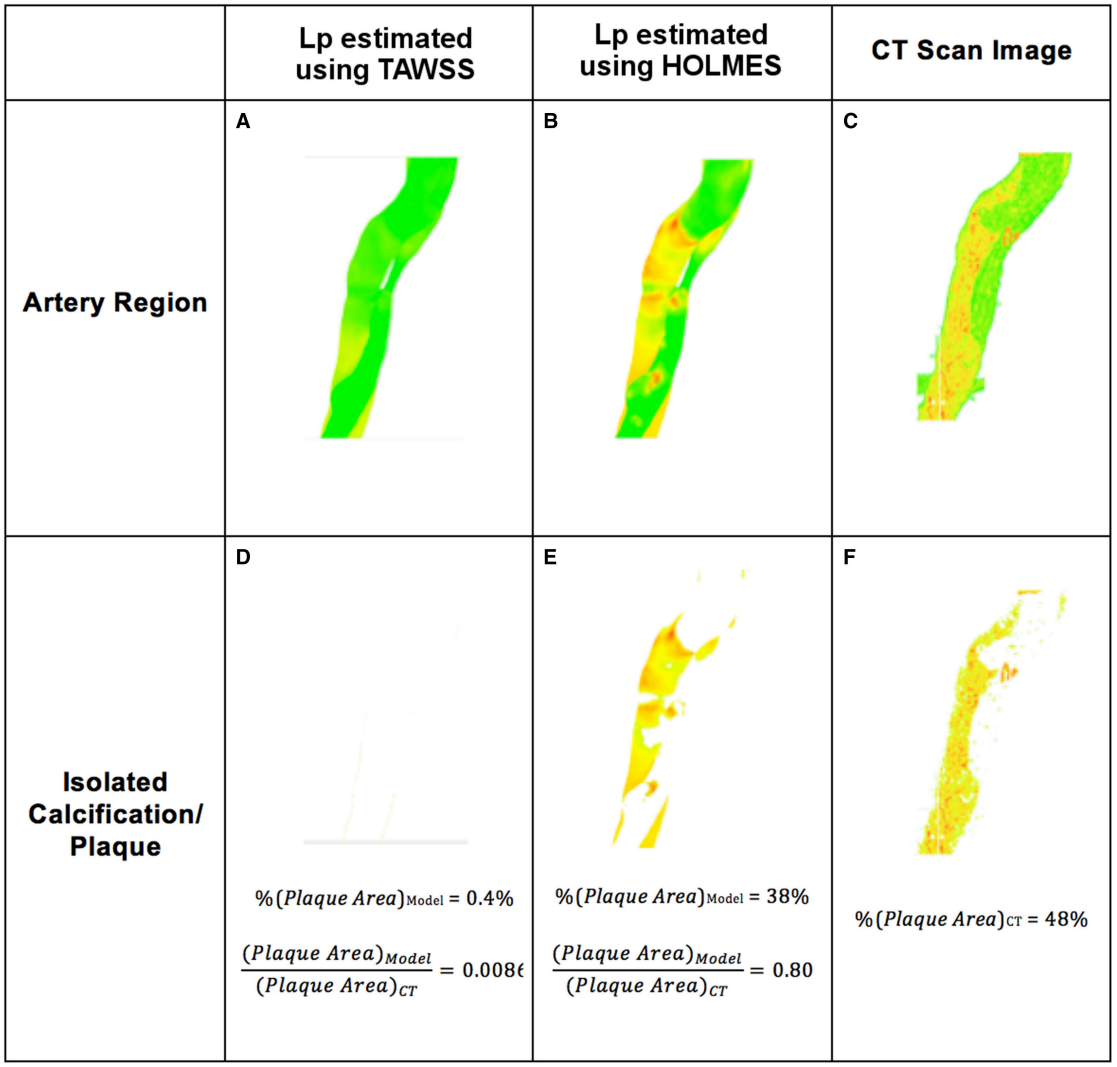
**Example of atherosclerotic plaque identification from simulations using hydraulic conductivity and comparison with 3D CT scans**. **(A,B)** Section of the artery showing plaques based on *L*_*p, lj*_; **(D,E)** Plaque extracted from **(A,B)**; **(C)** Section of the artery from CT scan; **(F)** Plaque extracted from **(C)**.

In addition to the clear qualitative improvement in the correlation between the CT scans and *L*_*p, lj*_ and *J*_*v, lj*_ distributions, we utilized a simple measure based on plaque areas, as shown in Figure 9 for the descending aorta, a key region in the case of AD (as explained in Section Estimating Plaque Location and Metrics Evaluation).

When comparing *L*_*p, lj*_ values in specific regions of the aorta, using the results shown in Figure 5, the calcified regions predicted by the model overlapped 80% of the calcified regions observed in the CT scans for the HOLMES model. No calcification was predicted using TAWSS. The next step was to analyze the results of *J*_*v, lj*_ (given its stronger relationship with the LDL flux).

**Figure 10:**
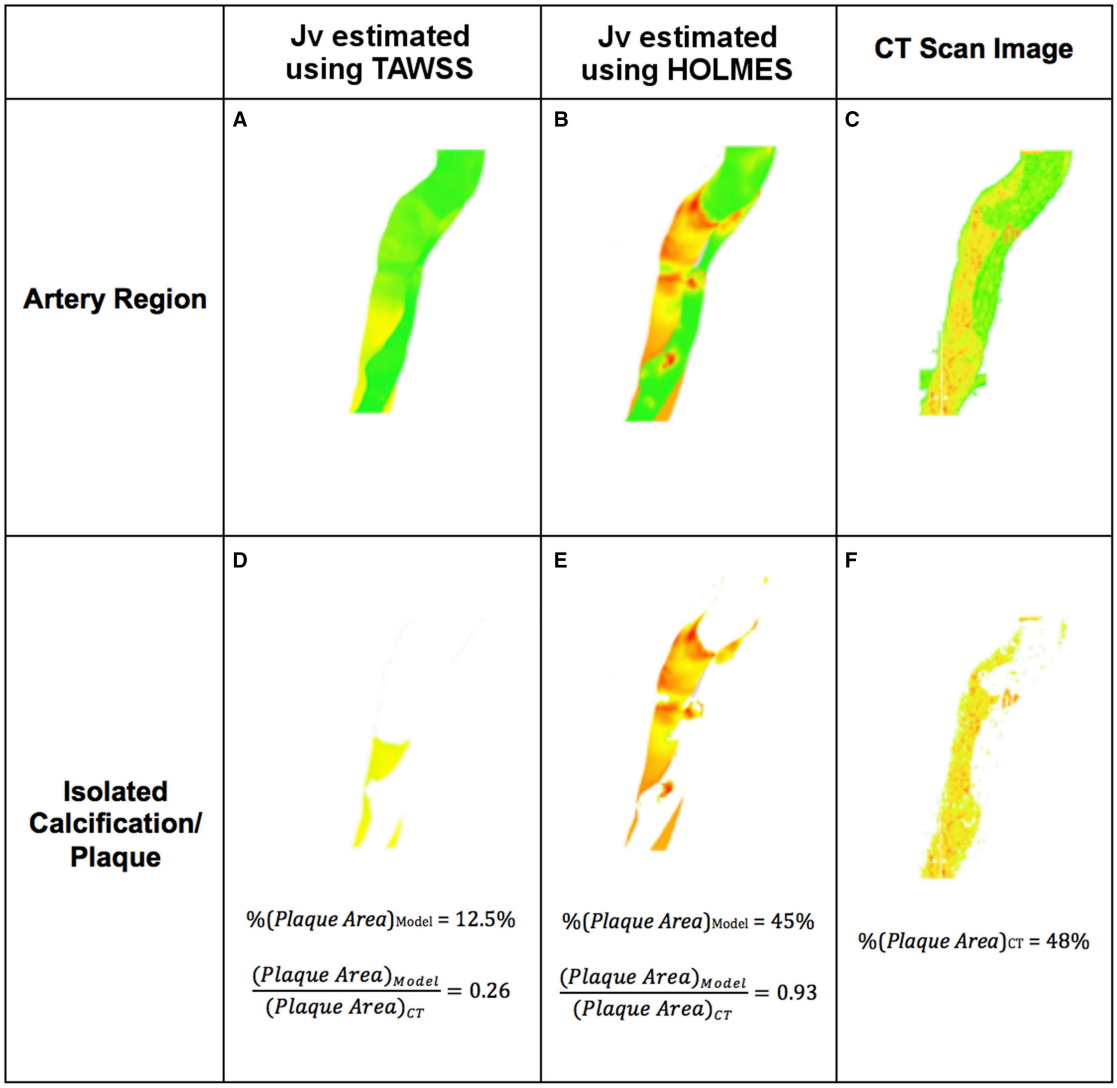
**Example of atherosclerotic plaque identification from simulations using solute flux and comparison with 3D CT scans**. **(A,B)** Section of the artery showing plaques based on *J*_*v, lj*_; **(D,E)** Plaque extracted from **(A,B)**; **(C)** Section of the artery from CT scan; **(F)** Plaque extracted from **(C)**.

Figures 10A,C show *J*_*v, lj*_ in this region calculated with TAWSS and HOLMES, respectively and Figures 10B,D show the calcified region only. Figures 10E,F show the equivalent figures for the CT scans. Comparing the proportional area of the calcified region between the two cases, 26 and 93% of the regions overlap for TAWSS and HOLMES, respectively. When calculated using HOLMES in the selected region (Region 3 in Figure 2), 45% of the visible arterial wall is predicted to be prone to calcification, compared to 48% of the visible arterial wall observed in the CT scan image. Applying the same analysis using HOLMES to the whole aorta predicts between 80 and 95% of overlapping. This analysis supports the *potential* predictive power of the proposed model.

From a hemodynamics point of view, the heterogeneous distribution of calcification/plaque formation will be influenced by disturbed flow. Wall shear indices such as TAWSS and OSI capture only partial aspects of the complex flow fields. The wall shear stress vector has both direction and magnitude at each moment throughout the cardiac cycle. TAWSS captures the average magnitude, but contains no information about directionality. OSI characterizes the variability in the directionality. However, a number of indices (“emerging” multidirectional predictors, as defined by Gallo et al., 2016) have been proposed and some of these might have potential as predictors of atherogenesis. Peiffer et al. (2013b) proposed the transverse wall shear stress, which is the temporal average of the component of the wall shear stress vector that is perpendicular to the time average wall shear stress vector. Morbiducci et al. (2015) defined the preferential direction as being relative to the direction of the vessel centerline and similar considered the temporal average, producing an additional index based on the temporal average of the ratio of these two components. Arzani and Shadden (2016) also introduced backward wall shear stress, considering the average of the negative instances of wall shear stress in the direction opposite to the centerline. In future studies, it will be important to explore the efficacy of these new indices.

In this paper, we have exclusively focused on the “established” predictors, for which the links between endothelial cell behavior and haemodynamics have been shown in the relevant literature as described above.

In the work presented here, the use of patient-specific data (including invasive haemodynamic measurements) and BCs, inclusion of wall motion and use of a non-Newtonian blood viscosity model are likely to improve the estimates of these hemodynamic variables and thereby increase the likelihood of an improved correlation. Importantly, as evidenced by Figures 4, 7, the interpretation of the role of these variables individually was unable to predict distributions of atherogenesis/calcification. It was only through a combination of HOLMES, a compound shear index, a non-Newtonian fluid viscosity and a sophisticated simulation model that this was achieved. In general, when extracting information related to values calculated at the wall, motion is extremely important. In this particular case and as shown previously (Alimohammadi et al., 2015b), simulating wall motion is key, since the dynamic interactions between the intraluminal pressure-gradient, the vessel wall elasticity and the intimal flap motion play a critical role in accurately predicting haemodynamics in the false lumen. Moreover, in healthy aortae, rigid wall simulations have been reported to produce somewhat comparable distributions of WSS, albeit with overestimated magnitude (Brown et al., 2012; Reymond et al., 2013). In dissected aortae, the differences between the results of rigid wall (CFD only) and FSI simulations are significant (Alimohammadi et al., 2015b), which has a clear impact in the interpretation of the effect of mechanical stimuli on endothelial behavior in this case. These differences are enhanced by the complex, intertwining lumina, in which even small motion variations have a decisive effect. Hence, the use of FSI, while more time-consuming and technically challenging, is important.

### Limitations

One limitation of the fluid dynamics simulation is the absence of the visceral arteries, which would alter the flow in the descending aorta. As previously stated, it was not possible to resolve these vessels from the CT scans, so they were omitted. Nonetheless, even in their absence, we believe the usefulness of the model was demonstrated.

A patient-specific inflow waveform was not available for the present study, and as such, a waveform from the literature was selected from a patient suffering from a similar type-B AD. The absence of a patient-specific inflow reduces the specificity to the patient of the present results however, given that the boundary conditions were tuned to patient-specific values using the same inflow, the hemodynamic environment predicted by the simulations is expected to be an appropriate representation of a patient with type-B AD. The flow waveform at the inlet was applied as a uniform velocity, rather than mapping to parabolic or Womersley profiles. Whilst accurate axial velocity profile, as extracted using pcMRI can provide improved predictions of characteristics such as helical flow (Morbiducci et al., 2013), it is not clear that parabolic or Womersley profiles offer any improvement over a uniform velocity (Marzo et al., 2009; Campbell et al., 2012; Morbiducci et al., 2013).

The isotropic hyperelastic model of Raghavan and Vorp (2000) is a simplification of the true aortic wall properties, which are known to be anisotropic (Gasser et al., 2006). This model has been shown to provide improved predictions of stress distributions compared with a linear elastic model, but underestimates peak stresses relative to anisotropic models (Roy et al., 2014). However, it is important to note that wall stresses are not explicitly evaluated in the present study; additionally, fibrosis in the vessel wall alters wall properties over time in AD, and is thus highly patient-specific. Furthermore, wall motion was not captured as part of the clinical data collection. More detailed imaging as well as experimental data on vessel wall properties in AD is necessary for better, patient-specific simulations. Given these constraints, although we acknowledge that more complex models of vessel wall properties have been applied in AD and that this might result in local variations, in this case these are unlikely to yield improved accuracy in what is our ultimate goal, the quantification of their effect on hemodynamic parameters for individual patients.

Another limitation of this approach, which is fairly consistent throughout the related literature is the use of “visual maps,” in order to establish qualitative correlations between the *in vivo* data and the simulation results. Recent studies address the issue of quantification of these type of results by using statistical methods to establish quantitative correlations and statistical significance of the variables analyzed (simulated) with respect to plaque location, obtained from *in vivo* data (Morbiducci et al., 2015; Gallo et al., 2016). This is clearly a welcome development that we will be keen to use for future, multi-patient studies. As it has been presented here, the focus of this paper is on the use of multi-scale modeling and simulation tools and the development of interpretable, physiologically-based metrics to understand plaque location.

The results presented in this paper when using the multi-criteria, patient-specific, multi-scale complex framework described here, compare well, qualitatively, to *in vivo* data and although there is no perfect match, this is to be expected, when considering the number of assumptions, simplifications and limitations as described (please see above). Moreover, as for patient-specific data, only anatomical information was fed into the model and hemodynamic variables were calculated based on invasive pressure measurements in order to properly characterize the flow, as described in Alimohammadi et al. (2014). Apart from the hemodynamic calculations previously described, it is important to mention that the authors have been extremely careful to inform the model with data and values taken straight from the literature, so, there is no unique estimation of parameters or “fitting” apart from appropriate characterization of flow variables, for the simulation presented here. It remains a striking feature of this approach that even taking this into account; the *correspondence* between observed and simulated calcified/atherosclerotic regions is high, can be explained and appears to be a marked improvement upon other standard methods reported in previous studies. It also offers a coherent, mechanistic explanation that is able to shine some light on combined mechanisms responsible for the location of atherosclerotic/calcification areas and to interpret them together and simultaneously. Although the focus of this paper is on the use of multi-criteria and multi-scale mathematical modeling to understand atherosclerosis and results are shown for one patient only, it is important to say that preliminary and very encouraging results from a different anatomical site with a simpler model, but also following a mechanistic approach have been published in Alimohammadi et al. (2015a). Equally, results from other simulations currently performed by our group (not shown here) using the approach developed in this work for different arteries, show similar effectiveness than the one discussed here, in terms of identification of calcification/atheroprone areas. We acknowledge nevertheless the need to test this approach in a small cohort of patients, taking into account the limitations and new developments described above. This is work that is already under way.

## Conclusions

In the present study, we have presented a mechanistic, mathematical model of endothelial mechanotransduction to understand plaque location/calcification. The model is tested on a patient-specific case for which *in vivo* measurements were obtained at University College Hospital and a patient-specific biomechanical model produced (Alimohammadi et al., 2015a) and attempts to provide a clear, multi-factorial metric for plaque location, with strong physiological meaning and interpretation. The results from this model compared favorably with *in vivo* data and outperformed other well-established indices currently used in the literature. The model used an advanced FSI simulation, comprising patient specific dynamic outlet boundary conditions and non-Newtonian blood viscosity. This was coupled with an established model for atherogenesis in order to investigate the roles of various hemodynamic parameters on the development of calcified regions in the aortic wall.

As an input to the endothelial permeability model relating to shear stress, we hypothesized that regions of oscillatory, low magnitude shear stress would be susceptible to calcification, due to the known connection to increased permeability. We therefore, proposed the compound HOLMES shear index, which includes both magnitude and oscillatory characteristics and will thus emphasize oscillatory, low magnitude shear stress and found that it considerably improved the predictive power of the model over TAWSS-based analysis.

The role of the complex characteristics of the hemodynamics near the wall was investigated by hypothesizing that elevated blood viscosity near the wall would limit the convection of plasma into the vessel wall, and vice versa. It was found that when the spatially varying blood viscosity at the wall as estimated using an empirical non-Newtonian viscosity model, was used in the endothelial permeability model, the predicted *L*_*p, lj*_ and *J*_*v, lj*_ distributions bore increased resemblance to the observed regions of calcification.

This work provides a good example of the use of multiscale mathematical modeling to understand physiology. The promising results obtained from this approach warrant further investigation. Next steps will include studies of a larger number of patients to enable comparisons amongst patients and potentially statistical analyses, in order to investigate in detail the predictive power of the model.

## Author contributions

MA and CP conceived the study and carried out simulations and wrote the manuscript. OA acquired the clinical data and VD conceived the study and wrote the manuscript.

## Funding

EPSRC grant “Personalised Medicine Through Learning in the Model Space” (grant number EP/L000296/1). Leverhulme Trust Senior Research Fellowship “Exploring the Unknowable Using Simulation: Structural Uncertainty in Multiscale Models” (Fellowship number RF-446 2015-482).

### Conflict of interest statement

The authors declare that the research was conducted in the absence of any commercial or financial relationships that could be construed as a potential conflict of interest.
